# Potential multiple disease progression pathways in female patients with Alzheimer’s disease inferred from transcriptome and epigenome data of the dorsolateral prefrontal cortex

**DOI:** 10.1371/journal.pone.0313733

**Published:** 2025-03-18

**Authors:** Kousei Honda, Akinori Awazu

**Affiliations:** 1 Graduate School of Integrated Sciences for Life, Hiroshima University, Higashihiroshima, Hiroshima, Japan; 2 Research Center for the Mathematics on Chromatin Live Dynamics, Hiroshima University, Higashihiroshima, Hiroshima, Japan; Jhargram Raj College, INDIA

## Abstract

Late-onset Alzheimer’s disease (AD) is a typical type of dementia for which therapeutic strategies have not yet been established. The database of the Rush Alzheimer’s Disease study by the ENCODE consortium contains transcriptome and various epigenome data. Although the Rush AD database may contain a satisfactory amount of data for women, the amount of data for men remains insufficient. Here, based on an analysis of publicly available data from female patients, this study found that AD pathology appears to be nonuniform; AD patients were divided into several groups with differential gene expression patterns, including those related to cognitive function. First, cluster analysis was performed on individuals diagnosed with “No Cognitive Impairment (NCI),” “Mild Cognitive Impairment (MCI),” and “Alzheimer’s Disease (AD)” stages in clinical trials using gene expression, and multiple substages were identified across AD progression. The epigenome data, in particular genome-wide H3k4me3 distribution data, also supported the existence of multiple AD substages. However, *APOE* gene polymorphisms of individuals seemed to not correlate with disease stage. An inference of adjacency networks among substages, evaluated via partition-based graph abstraction using the gene expression profiles of individuals, suggested the possibility of multiple typical disease progression pathways from NCI to different AD substages through various MCI substages. These findings could refine biomarker discovery or inform personalized therapeutic approaches.

## Introduction

Alzheimer’s disease (AD) is the most common cause of dementia, accounting for 60–70% of dementia cases in older adults [1,2]. AD can be classified into the familial type, which is strongly influenced by genetic factors, and the late-onset (sporadic) type, which is strongly influenced by aging and environmental factors, with the latter accounting for the majority of AD cases [3]. No treatment or preventive methods for AD have yet been established because the fundamental pathogenic mechanism of sporadic AD remains unclear.

In recent years, transcriptome and genome-wide analysis data of nucleotide polymorphisms have been collected from patients with AD, and various differentially expressed genes (DEGs) and patient-specific SNPs have been identified in healthy individuals and patients with AD [4–6]. For example, the DEGs inferred by recent transcriptome data analyses by several researchers were listed in the recent literature [6] Notably, these characteristics are not always seen equally across all studies. One reason for this is the variations in technical factors among studies. However, the diversity in molecular conditions and gene expression patterns in the brain among patients with AD could also be a major contributor [7–11].

Recently, plaques of abnormally aggregated amyloid β (Aβ) were considered to be closely related to AD [12–15]. However, other factors such as the abnormal aggregation of tau protein [16], expression of apolipoprotein E variant *APOE4* [17], herpes virus and coronaviruses (including SARS-CoV2) infections [18–21], diabetes [22,23], brain inflammation [24], oxidative damage in cells [25–27], endoplasmic reticulum and ciliary dysplasia [28–31], myelin dysplasia [32,33], and retrotransposons activities [34–36] have also been reported to influence the onset and progression of AD. Owing to the involvement of the aforementioned factors, the causes, induced gene expression variations, and symptoms as the results of the gene expression variations of AD are thought to vary widely from patient to patient. To this end, variations in symptoms and disease progression rates among patients [37–40], variations in activities of key proteins such as PAPP-A and KDM6A [41–43], and the patients-dependent 4 distinct spatiotemporal trajectories of tau pathology [44] have been previously reported.

Thus, AD progression is expected to exhibit various disease states. Classifying and characterizing these disease states and progression pathways from the transcriptome state, playing the background role of pathologies, is necessary to enable appropriate state-dependent treatment. Notably, transcriptome states are always regulated closely by and regulate epigenome states. Thus, in addition to a comparison of the features of the entire transcriptome, a comparison of the entire epigenome status among patients should be useful for such classification and characterization of disease diversity.

The database of the Rush Alzheimer’s Disease study by the ENCODE consortium (https://www.encodeproject.org/) [45,46] contains transcriptome and four epigenome data such as histone modifications (H3K27ac, H3K27me3, and H3K4me3) and CCCTC-binding factor (CTCF) binding site distribution from individuals in the “No Cognitive Impairment (NCI),” “Mild Cognitive Impairment (MCI),” and “Alzheimer’s Disease (AD)” groups that are available for unlimited public use (termed Rush AD database thereafter). Therefore, analyzing these publicly available data of patients with different cognitive impairment stages is expected to elucidate the diversity in the onset and progression of late-onset AD in more detail.

Note that the Rush AD database seemed to contain an insufficient amount of data for males since the number of male patients providing data in NCI, MCI, and AD groups was 14, 4, and 7, respectively. However, the database seemed to contain a satisfactory amount of data for women, where the number of patients in NCI, MCI, and AD groups was 25, 29, and 28, respectively Therefore, in this study, the classification of disease status and AD progression pathways was performed using transcriptome and epigenome data of female patients in the Rush AD database as a first step of the analysis of the diversity of patients with AD using multiomics data. Of course, some features are known to be different between male and female patients, for example, the disease progression tends to be faster in male than in female patients [42,43]. However, besides the abovementioned features, our findings are expected to be useful for understanding symptoms in both female and male patients.

## Materials and methods

### Ethics statement

This study utilized publicly available data from the ENCODE database, specifically the Rush AD study. As this research involved the analysis of de-identified, publicly accessible data, it did not require approval from an Institutional Review Board (IRB) or ethics committee. No additional informed consent was necessary as the data were already collected and made available for research purposes. We adhered to all relevant ethical guidelines for the use of public data in research.

### Fundamental features of the analyzed transcriptome and epigenome data

RNA sequencing (RNA-seq) data, read count data and the data normalized as transcript per million (called TPM data), of the dorsolateral prefrontal cortex of female individuals belonging to the NCI, MCI, and AD groups were available from the Rush AD database. In this dataset, the number of RNA-seq data of individuals in the NCI, MCI, and AD groups were 25, 29, and 28, respectively (Table 1, and S1a Table). In the present analysis, the data of genes with average expression levels (TPM) in patients with NCI, MCI, and AD smaller than 0.1, and those of spike-in-RNAs were eliminated.

**Table 1:** Summary of the individuals included in this study. See S1a Table for more details.

Clinical stage	No Cognitive Impairment (NCI)	Mild Cognitive Impairment (MCI)	Alzheimer’s Disease (AD)
# of individuals	25	29	28
Individual index	N1 – N25	M1 – M29	A1 – A28

The data provided by the Rush AD study in ENCODE does not publish information on RNA quality such as the postmortem interval of samples and RIN values. However, according to ENCODE standards, no specific alerts regarding the quality of the RNA-seq data used in this study were provided. Thus, the quality of the data was assumed to be guaranteed. The set of genes whose expression changes between patients with NCI and AD and among patients with NCI, MCI, and AD, involved many genes identified as AD risk genes in previous studies. This further supported the lack of data bias that would contradict previous studies.

To examine the quality and bias of analyzed data, we estimated one of the size factors of the RNA-seq data, that is, the median of the ratios (MOR) defined in the recent literature for DESeq [47], using read count data (S1a Table). The ratio [standard deviation of MOR over individuals]/[average MOR over individuals] was 0.1075. Similarly, we evaluated the first and third quartiles of the ratio (1OR and 3OR) between the average and standard deviation, which were 0.1147 and 0.1006, respectively. These results suggested that the size factor would not considerably input bias among samples or affect the comparison of either the read count data or TPM values.

When examining the significance of the difference in gene expression levels between individuals aged < 85 years and those > 90 years, we did not detect any genes exhibiting a false discovery rate (FDR) <  0.1 (likelihood ratio test using DESeq2 software [48] and two-tailed *t*-test) (S1b Table). This sugges*t*ed that the variations in gene expression levels among the NCI, MCI, and AD groups was not due to the influence of age but rather due to differences in clinical stages.

In addition to RNA-seq data, the file of the bigwig format data of fold change over control (FC) on genome-wide histone modifications (H3K4me3, H3K27ac, and H3K27me3) and CTCF binding site distribution (binary file) obtained through chromatin immunoprecipitation sequencing (ChIP-seq) were acquired from the Rush AD database for 39 individuals: 11, 11, and 17 individuals from the NCI, MCI, and AD groups, respectively (S1a and S1c Tables). In this study, the regions with FC >  5 were defined as the regions of peak intensities of these epigenome marks according to the literature of ChIP-seq analysis [49].

### Overview of analyses

This study considered patient diversity by assessing the deviation from healthy individuals and the diversity of disease progression. For this purpose, we first searched for differentially expressed gene sets between healthy individuals and patients. From these gene sets, we focused on those that contained the largest number of genes and that could distinguish healthy individuals from patients using cluster analysis. Second, we evaluated the variety in the expression patterns of those gene sets between patients to show the existence of diverse conditions and subgroups within patients. The existence of these subgroups was also investigated by analyzing epigenomic data and *APOE* polypholism among individuals. Third, the relationship between subgroups and the structure of the disease progression pathway was predicted based on the similarity in gene expression between subgroups.

### Statistical analyses of transcriptome data

Transcriptome data were statistically analyzed as follows: The likelihood ratio test using the DESeq2 software [48], one of the most familiar tests for estimating DEGs from read count data. We performed this test to extract the differentially expressed genes (DEGs) among two or three patient groups from RNA-seq read count data. Here, the count data is normalized by the method implemented by DESeq2 to evaluate DEGs [48]. Additionally, to extract DEGs among three patient groups from TPM data, we performed the analysis of variance (ANOVA), which is the most familiar test for evaluating differences in characteristic values among three or more groups. We also performed Two-tailed t-tests to extract the DEGs between two patient groups from TPM data.

Two-tailed *t*-tests and ANOVA were performed using Python (version 3.9.16). All *t*-tests in this study were Welch’s *t*-tests. The false discovery rate (FDR) was estimated through a set of P-values using the Benjamini–Hochberg procedure [50] implemented in MultiPy (https://puolival.github.io/multipy/) [51]. The search for DEGs via the likelihood ratio test using DESeq2 and the calculation of P-values for the Fisher’s exact test were performed using R (version 4.1.3).

This study aims to explore the potential diversity hidden in AD symptoms and their progression and the possible factors that characterize them. Therefore, we prioritized the possibility of exploring more potential factors over suppressing the occurrence of false positives. Therefore, when statistical analysis of gene expression levels was required, such as testing the difference between groups in expression levels or ANOVA, the FDR threshold indicating significance was set to 0.1. This condition is the same as that used in numerous previous studies of human medicine (for example see [52,53]).

### Nomenclature of threshold values for P-values in statistical test of transcriptome data

As mentioned subsequently, this study performed cluster analyses to classify individuals using the expression levels of each gene of the individuals. The gene set used in the cluster analysis is selected based on the relationship between the P-value obtained from the test of the difference in expression levels between the focused individuals groups (likelihood ratio test using DESeq2 [48], ANOVA, or t-test) and a given threshold. In this study, various combinations of individual groups were analyzed using various testing methods. Therefore, numerous thresholds related to the test appeared.

Here, we introduce a nomenclature for each threshold according to the combination of subject groups and various testing methods. Each threshold value was written in P^[E]^_[S]_, where [E] indicates the index of the test and [S] denotes the index of a combination of compared groups. In this study, [E] was given by “D” for the likelihood ratio test using DESeq2, “A” for ANOVA, and “t” for t-test. The subscript [S] was formed by the combination of indices of individual groups where indices for the groups of NCI, MCI, and AD individuals were given by “N,” “M,” and “A,” respectively. For example, the threshold value of the P-value obtained by ANOVA among NCI, MCI, and AD individuals was written as P^A^_NMA_.

When [S] was formed by two indices, such as XY, the order of X and Y involved the magnitude relationship between the mean expression levels of a focused gene in groups X and Y. For example, the threshold value for the P-value obtained by t-test of the expression levels of a focused gene between NCI and AD individuals was written as P^t^_NA_ when the mean TPM value in AD individuals was larger than that in NCI individuals, and as P^t^_AN_ when the mean TPM value in NCI individuals was larger than that in AD individual.

### Cluster analyses of individuals based on transcriptome data

In this study, individuals are classified by cluster analysis using the transcriptome data of the individuals. For cluster analyses, instead of raw transcriptome data, the values of X =  log_2_ (TPM +  1) – C were used, where C was chosen to satisfy the average of X over individuals =  0 for each gene. iDEP.96 (http://bioinformatics.sdstate.edu/idep96/) was used for hierarchical clustering [54]. In hierarchical clustering, the group-average method, was used to create a dendrogram, using the correlation coefficient as the distance. If the cluster formed by the first branch of hierarchical clustering satisfied the following criteria for a certain group (NCI, MCI, and AD), then that cluster was regarded as a cluster including significantly more individuals belonging to that group. These criteria were as follows: i) containing more than 2/3 of the individuals belonging to that group, and ii) Fisher exact test resulting in a P-value <  0.05. Further, k-means cluster analysis was performed using the Python package scikit-learn (version 1.0.2) [55]. The number of clusters in each k-means cluster analysis was determined using the elbow method.

Additionally, the construction of UMAP was performed using sc.tl.pca() [55] with default parameters, scanpy.pp.neighbors() with “n_neighbors” =  30 [56], and the scanpy.tl.umap() functions with n_components =  3 [56] of scanpy (version 1.9.3) according to the literature on basic workflows for clustering analysis using Scanpy (https://scanpy-tutorials.readthedocs.io/en/latest/pbmc3k.html). All principal components were used for clustering using the scanpy.pp.neighbors() function. The obtained results were qualitatively independent of the parameters of these applications.

### Epigenomic data analysis

Rush AD database also included four ChIP-seq data on the genome-wide distribution of epigenomic markers such as histone modifications (H3K4me3, H3K27me3, and H3K27ac) and CTCF binding site distribution for approximately half of all individuals (S1a Table). Hence, to classify these individuals based on features other than transcriptome, we evaluated the similarity of such epigenomic features among individuals using Jaccard coefficients of the genome-wide distribution of previously defined peak regions of epigenome status.

The genome-wide similarity of each epigenetic state between individuals was evaluated using the Jaccard index as follows: the bigwig formatted files (binary files) of fold change over control of these epigenome markers were acquired from individuals whose ChIP-seq data of H3K4me3, H3K27ac, H3K27me3, and CTCF were provided in the Rush AD database. From each bigwig format data, the file of bedgraph format data (text file) was obtained using bigWigToBedGraph with default parameters (bigWigToBedGraph [Input file name of bigwig format data] [Output file name of bedGraph format data]) [57]. A file of bed format data of peak regions was obtained from the bedgraph format data following the removal of regions with FC <  5 where epigenome marker accumulation was considered not significant. Here, the threshold value 5 was determined, referring to the literature of ChIP-seq analysis [49]. The Jaccard coefficients between each pair of individuals were estimated using the jaccard function of Bedtools (version 2.31.0) with default parameters (bedtools jaccard -a [First input file of bed format data] -b [Second input file of bet format data]). The Jaccard coefficient represents the degree of overlap in the peak distribution of epigenomic markers between individuals, i.e., similarity of the epigenomic state between individuals.

The density of each epigenomic marker on each chromosome was estimated using the file of bed format data for peak regions of the epigenome marker as follows: the total length of peak regions of each epigenome marker in each chromosome could be obtained by each file of bed format data. Additionally, the total length of each chromosome could be obtained from human genome data such as GRCh38 (https://www.ncbi.nlm.nih.gov/datasets/genome/GCF_000001405.26/). Thus, the density of each marker on each chromosome was calculated according to the following equation: [Total length of peak regions in the chromosome obtained from the bed formatted file (bp)]/[Total length of chromosome obtained from GRCh38 (bp)].

### Cluster analyses of individuals based on epigenome data

In this study, individuals are also classified by cluster analyses using the epigenome data of the individuals. The dendrogram showing the relationship between individuals based on the epigenome state was created as follows: first, for each individual, we consider a vector whose components were the Jaccard coefficients of this individual with other individuals. Second, the hierarchical clustering of individuals was performed based on the group average method using these vectors. Here, the hierarchical clustering was performed using the Python package scipy (version 1.13.1: https://scipy.org/).

The optimal number of clusters for hierarchical clustering of individuals based on the Jaccard coefficients of the genome-wide distributions of each epigenome marker was evaluated using the elbow method. The calculation of P-values for the Fisher’s exact test were performed using R (version 4.1.3).

### Analysis of correlation between the changes in expression level and epigenome status of promoter for each gene

In this study, the correlation between the expression level of each gene and the epigenetic marker density in the promoter region was also calculated using the obtained data of epigenetic marker peak regions (file of bed format data) and transcriptome (TPM) of individuals as follows. First, we defined the upstream 1 kbp of each gene as the promoter and created a bed format data file (promoter.bed) for those regions by referring to the annotation file gencode.v29.annotation.gtf (https://www.gencodegenes.org/human/release_29.html). Here, gencode.v29.annotation.gtf is the same as that used for the quantification of RNA-seq in Rush AD.

Next, we used the intersection function of Bedtools to obtain the overlapped region between epigenetic marker peak regions of each individual and the promoter region of each gene (bedtools intersect -a [File of bed format data of epigenome marker peak regions in an individual] [promoter.bed]). Then, we defined [the length of this overlapped region (bp)]/ [1000 (bp)] as the epigenetic marker density in the promoter region of each gene in each individual.

The correlation between the expression level (TPM) data of each gene and the data of epigenome marker density in its promoter in the focused individuals group was estimated by Pearson correlation between two data in that group.

### Analysis of *APOE* gene polymorphisms

The identification of the *APOE* genotype of each individual was also performed using the RNA-seq read data of individuals in the Rush AD database as follows. RNA-seq sequence read data (fastq format data) of patients with NCI, MCI, and AD were acquired from the Rush AD database. Fastp (https://github.com/OpenGene/fastp) was used to remove low-quality reads and trim adapter sequences. Following fastp processing, the reads were mapped to the CDS sequence of the *APOE* exon 4 of GRCh38 using Hisat2 (https://daehwankimlab.github.io/hisat2/), and results were obtained as a bam format file. Variant calling was performed using the obtained bam format file and CDS sequence data of the GRCh38 *APOE* exon 4 using bcftools (https://github.com/samtools/bcftools). After filtering out low-quality calls (QUAL < 20), genotypes were determined based on the type of mutation and allele frequency.

### Enrichment analysis

Enrichment analysis was performed using Metascape (v3.5.20240101) (https://metascape.org/gp/index.html#/main/step1) [58]. The biological process terms in Gene Ontology were the main focus of this study.

### Construction of an adjacency network among substages

To reveal the relationships among different disease stages (substages), the adjacency network among them was estimated via PAGA [59] using the gene expression data of each individual. Each group at each substage was regarded as a partition in PAGA. PAGA can generate a graph of substages in which the edge weights represent connection confidence. PAGA was performed using sc.tl.pca() [55] with default parameters, scanpy.pp.neighbors() with “n_neighbors” =  2 [56], and the scanpy.tl.paga() functions [59] of scanpy (version 1.9.3) according to the literature for trajectory inference using transcriptome data (https://scanpy-tutorials.readthedocs.io/en/latest/paga-paul15.html). All principal components were used for clustering using the scanpy.pp.neighbors() function. Additionally, to extract the essential network among substages, the value of the parameter “n_neighbors” of the function scanpy.pp.neighbors() was set as two, which is the minimum value in the range of this parameter. Thus, a network with nonredundant connections among substages was expected to be obtained.

## Results

### Cluster analysis of differentially expressed gene sets in the majority of patients with MCI

First, cluster analyses of all individuals were performed based on expression levels, where only a group, including the majority of MCI patients, was found to form a specific cluster, as follows.

**Fig 1:**
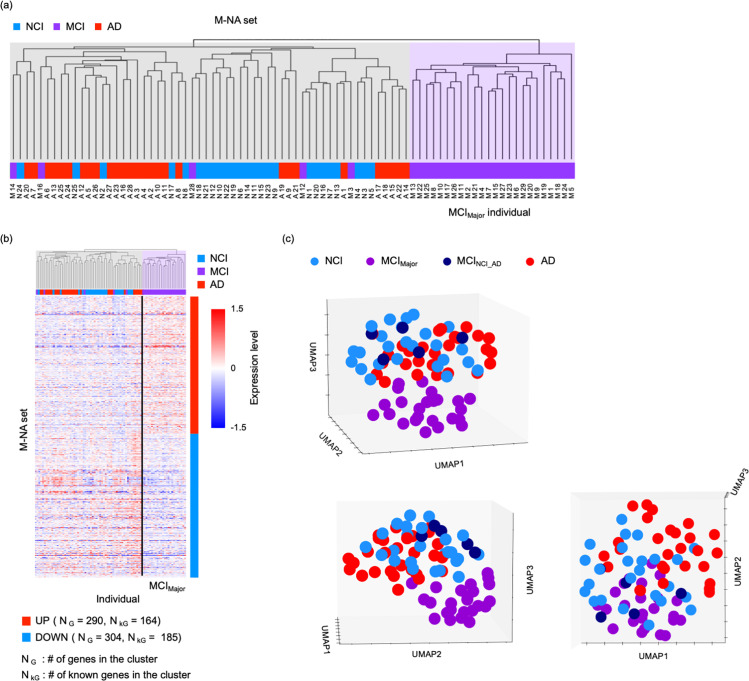
Results of the hierarchical clustering of individuals and of UMAP obtained using the M-NA set. Result of the hierarchical clustering of individuals when using the M-NA set (P^A^_NMA_ =  0.04) **(a)**. The expression level of each gene in the M-NA set for each individual was plotted **(b)** (see S2a Table for more details). The heatmap of the expression levels of each gene was described using X =  log_2_(TPM +  1) – C, where C was chosen to satisfy the average of X over individuals =  0 for each gene. MCI_Major_ individuals occupy the right region in the dendrogram. N_G_ and N_kG_ indicate the total number of genes and number of genes annotated as protein coding or noncoding RNA genes with higher (UP) and lower (DOWN) expression levels in MCI_Major_ individuals, respectively. Scatter plot of individuals in 3-dimensional space constructed using U-MAP with 1, 2, and 3 axes shown from various directions **(c)**.

To extract a set of DEGs among patients with NCI, MCI, and AD, we utilized the DESeq2 software [48] to perform the likelihood ratio test for each gene using read count data and ANOVA using TPM values (S1b Table). Consequently, we performed hierarchical clustering of individuals using genes with P-values below the threshold value P^D^_NMA_ or P^A^_NMA_ =  0.01, 0.02, 0.03, 0.04, and 0.05 (Fig 1, S1a–S1e Figs in Supplementary File). When P^D^_NMA_ or P^A^_NMA_ ≤  0.05, a cluster with significantly more patients with MCI (MCI cluster) and a cluster with patients with NCI, AD, and a small number of patients with MCI was formed via the first divergence of the dendrogram (P <  0.05, Fisher’s exact test; S1 Fig in Supplementary File). Additionally, when the P^A^_NMA_ was 0.04, a cluster comprising solely patients with MCI was formed through the first divergence of the dendrogram (Fig 1a). In contrast, we did not observe any clear separation between patients with NCI and those with AD, regardless of the P^D^_NMA_ and P^A^_NMA_ values.

Notably, we did not obtain any gene set when applying the P^D^_NMA_ and P^A^_NMA_ values that enabled the complete discrimination of patients with NCI, MCI, and AD. In addition, depending on the P^D^_NMA_ and P^A^_NMA_ values, the patients with MCI who deviated from the MCI cluster varied; however, 24 patients with MCI were always included in the MCI cluster (S1 Fig in Supplementary File). This suggests that the MCI cluster consisted of 24 patients with MCI, named MCI_Major_ individuals, whereas the 5 other patients with MCI (M3, M12, M14, M16, and M28) who tended to deviate from the MCI_Major_ individuals, were named MCI_NCI_AD_ individuals. We assumed that the MCI_Major_ and MCI_NCI_AD_ individuals were classified to the substages named MCI_Major_ and MCI_NCI_AD_, respectively.

Moreover, the set of genes with P-values of the above ANOVA ≤  P^A^_NMA_ =  0.04 was named the “M-NA set” as it significantly differed between the majority of patients with MCI from those with NCI and AD (Fig 1b, S2a Table). In a 3-dimensional U-map using the M-NA set, we clearly observed the deviation of the MCI_Major_ individuals from those at other substages, with MCI_NCI_AD_ detected in a mixed group of patients with NCI and AD (Fig 1c).

The classification result of patients with MCI into MCI_Major_ and MCI_NCI_AD_ individuals seemed to be independent of the age of individuals. For example, the numbers of patients over and under 90 years old in MCI_Major_ were 19 and 5, whereas those in MCI_NCI_AD_ were 3 and 2, respectively (P-value of the Fisher’s exact test =  0.5688; S1a Table).

### Functional features of genes in the M-NA set

We also considered the functional features of genes in the M-NA set. In this set, we obtained 594 genes (290 upregulated and 304 downregulated genes in MCI_Major_); of them, 349 genes (164 upregulated and 185 downregulated in MCI_Major_) were annotated (Fig 1b) as protein coding or noncoding RNA genes using Entrez [60]. Herein, both sets of upregulated and downregulated genes in MCI_Major_ contained some previously reported AD risk-associated genes; in particular, the upregulated gene set contained *CYP19A1, GSTM1, HSD11B1, IL10, MT-RNR1, PCK1, TRAK2, UBE2D1*, and *XBP1*, whereas the downregulated gene set contained *MT-ATP8* and *NME8* [61].

In addition, we performed an enrichment analysis of all genes, including those with higher and lower expression levels in MCI_Major_ individuals (S2b–S2d Tables). We identified that genes with higher expression levels in MCI_Major_ individuals were enriched in GO terms for the functions “response to lipopolysaccharide,” “response to molecule of bacterial origin,” “Toll-like receptor signaling pathway,” “regulation of MHC class II biosynthetic process,” “vitamin metabolic process,” “water-soluble vitamin metabolic process,” “chemotaxis,” and “taxis” (P-value <  10^-3^ and FDR <  0.5) (S2c Table). These functional features are characteristic of AD pathology; the lipopolysaccharide found in the wall of all Gram-negative bacteria was reported to play a role in causing AD [62,63], Toll-like receptors were found to participate in neuronal cell death by apoptosis [64,65], MHC class II-expressing cells were suggested to be involved in the degradation of neurons [66], vitamins were reported to affect the generation and clearance of Aβ [67], and microglia were shown to exhibit chemotaxis for Aβ clearance [68,69].

Meanwhile, we identified that genes with lower expression levels in MCI_Major_ individuals were enriched in GO terms for the functions “mRNA 5′-splice site recognition” and “mRNA cis splicing, via spliceosome” (P-value <  10^-3^ and FDR <  0.5) (S2d Table).

### Differentially expressed gene sets between patients with NCI and AD

In the following, we focused on differences between NCI and AD individuals that were not distinguished in the three-group comparison among NCI, MCI, and AD.

**Fig 2:**
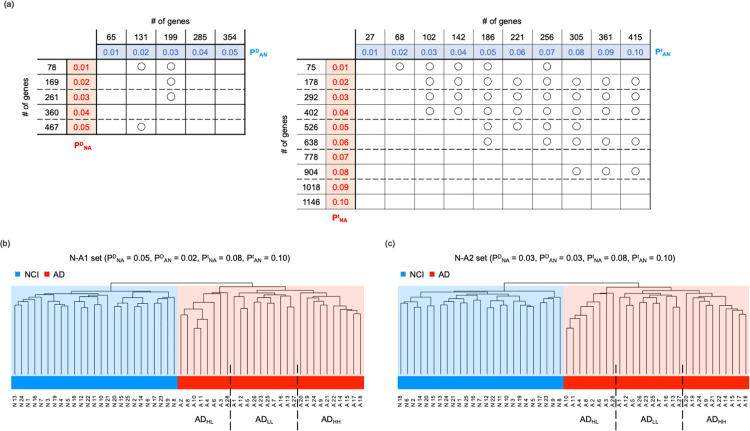
Phase diagrams of the hierarchical clustering of patients with NCI and AD, and the results of the hierarchical clustering of individuals in the N-A1 and N-A2 sets. Phase diagram of the results of hierarchical clustering when using genes where **p** ≤  P^D^_NA_ and **p** ≤  P^D^_AN_ (a, left), and that when using genes where **p** ≤  P^t^_NA_ and **p** ≤  P^t^_AN_ (a, right). Circles indicate the complete separation of patients with NCI from those with AD into different clusters at the first branch of the dendrogram. The dendrograms obtained using the N-A1 (b) and N-A2 sets (c) are shown. Broken lines indicate the boundary between the AD_HL_, AD_LL_, and AD_HH_ groups.

To extract a set of DEGs between patients with NCI and those with AD, we performed hierarchical clustering of individuals using the genes satisfying the following criteria: mean expression level (TPM) was higher in patients with AD than in those with NCI, with smaller P-values in the likelihood ratio test than the P^D^_NA_ threshold, or mean expression level (TPM) was lower in patients with AD than in those with NCI, with smaller P-values in the likelihood ratio test than the P^D^_AN_ threshold. Subsequently, we searched the sets of P^D^_NA_ and P^D^_AN_ values that divided the patients with NCI and those with AD into different clusters in the first branch of the dendrogram. Within the range P^D^_NA_ ≤  0.05 and P^D^_AN_ ≤  0.05, we identified the finite but narrow area of P^D^_NA_ and P^D^_AN_, P^D^_NA_ =  0.01 and P^D^_AN_ =  0.02, P^D^_NA_ =  0.01 and P^D^_AN_ =  0.03, P^D^_NA_ =  0.02 and P^D^_AN_ =  0.03, P^D^_NA_ =  0.03 and P^D^_AN_ =  0.03, and P^D^_NA_ =  0.05 and P^D^_AN_ =  0.02, where patients with NCI were completely separated from those with AD (Fig 2a). Thus, we obtained the largest set of genes separating the NCI and AD groups at P^D^_NA_ =  0.05 and P^D^_AN_ =  0.02 (S2a Fig in Supplementary File, S3a Table); this gene set was named the N-A_D1_ set. Notably, the number of genes with a lower mean expression level (TPM) in patients with AD than in those with NCI was larger when P^D^_NA_ =  0.03 and P^D^_AN_ =  0.03 than when P^D^_NA_ =  0.05 and P^D^_AN_ =  0.02 (S2b Fig in Supplementary File, S3b Table). Notably, this gene set contained all genes in other gene sets with P^D^_NA_ and P^D^_AN_ smaller than 0.03 that could separate the NCI and AD groups. Thus, we focused on the gene set obtained at P^D^_NA_ =  0.03 and P^D^_AN_ =  0.03 and named it the “N-A_D2_ set.”

We performed the same analysis using two-tailed *t*-tests to determine the P-values, defining the P^t^_NA_ and P^t^_AN_ instead of the P^D^_NA_ and P^D^_AN_, respectively. Herein, the *t*-test was generally better for detecting differences and was expected to identify more candidate genes in a gene set than the likelihood ratio test. Searching in the ranges P^t^_NA_ ≤  0.05 and P^t^_AN_ ≤  0.05, patients with NCI were completely separated from those with AD in 12 of 15 cases of P^t^_NA_ and P^t^_AN_ set for P^t^_NA_ ≤  P^t^_AN_ (Fig 2a). When we searched the wider range P^t^_NA_ ≤  0.1 and P^t^_AN_ ≤  0.1, patients with NCI were completely separated from those with AD in 37 of 55 cases of P^t^_NA_ and P^t^_AN_ set for P^t^_NA_ ≤  P^t^_AN_ (Fig 2a). We thus obtained the largest set of genes separating the NCI and AD groups at P^t^_NA_ =  0.08, and P^t^_AN_ =  0.1 (S2c Fig in Supplementary File, S3c Table). This gene set was named the “N-A_t_ set.”

Hierarchical clustering using a combination of the expression levels of all genes in the N-A_t_ and N-A_D1_ sets also separated patients with NCI from those with AD (Fig 2b, S3d Table). Conversely, the expression of all genes in the N-A_t_ and N-A_D2_ sets did not separate patients with NCI from those with AD. However, clustering using a combination of the expression levels of genes in the N-A_t_ set and a part of the N-A_D2_ set with low P-values completely separated patients with NCI from those with AD (Fig 2c, S3e Table). Such gene sets are expected to involve various typical differentially expressed genes between patients with NCI and those with AD. In this study, the gene set consisting of the N-A_t_ and N-A_D1_ sets, which could completely separate patients with NCI from those with AD, was named the “N-A1 set” (S3d Table), whereas the gene set consisting of the N-A_t_ set and maximum number of genes in the N-A_D2_ set with low P-values was named the “N-A2 set” (S3e Table).

### Patients with AD can be classified into three groups

In this subsection, we suggested AD patients were classified into three subgroups, as in the following.

**Fig 3:**
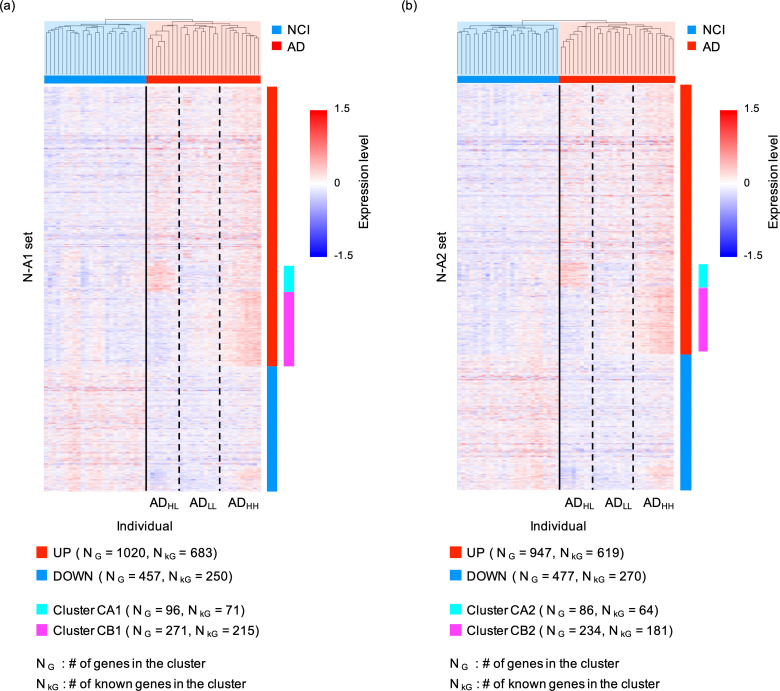
Results of the hierarchical clustering of individuals in the N-A1 and N-A2 sets and expression level of each gene. The dendrograms and expression level of each gene in the N-A1 set for each individual (a) (see S3d Table for more details) and those in the N-A2 set for each individual (b) (See S3e Table for more details) were plotted. The heatmap of the expression levels of each gene was described using X =  log_2_(TPM +  1) – C, where C was chosen to satisfy the average of X over individuals =  0 for each gene. Broken lines indicate the boundary between the AD_HL_, AD_LL_, and AD_HH_ groups.

First, from the results of the hierarchical clustering for patients with NCI and AD performed using the N-A1 set, we noticed the emergence of three subgroups of patients with AD (Figs 2b and 3a). Thus, we performed ANOVA on the expression levels of genes that were higher in patients with AD than in those with NCI among the three assumed subgroups (1020 genes). We identified 367 genes whose expression levels differed significantly (FDR <  0.1) among the 3 subgroups. Performing k-means cluster analysis of the expression levels of these genes in patients with AD, we identified two characteristic clusters: a cluster consisting of 96 genes the expression levels of which were low in only one subgroup (Cluster CA1) and another consisting of 271 genes the expression levels of which were high in only one subgroup (Cluster CB1) (Fig 3a).

Thereafter, we performed a similar analysis for patients with NCI. Based on the shape of the dendrogram, patients with NCI were divided into three or four subgroups (Figs 2b and 3a). However, when patients with NCI were divided into three subgroups, ANOVA revealed only nine genes with FDR <  0.1 among the three subgroups (S3d Table). Moreover, when patients with NCI were divided into four subgroups, ANOVA revealed only two genes with FDR <  0.1 among the four subgroups (S3d Table). Based on these values, the number of genes in clusters CA1 and CB1 was sufficiently large.

Collectively, these results suggested that the gene expression patterns in patients with AD, which can be classified into three types, were highly nonuniform. Patients with AD exhibiting high gene expression levels in both clusters, CA1 and CB1, were classified to the AD_HH_ subgroup (named AD_HH_ individuals), those exhibiting low gene expression levels in both CA1 and CB1 were classified in the AD_LL_ subgroup (named AD_LL_ individuals), whereas those exhibiting high gene expression levels in cluster CA1 and low gene expression levels in cluster CB1 were classified in the AD_HL_ subgroup (named AD_HL_ individuals).

Hierarchical clustering of patients with NCI and AD using the N-A2 set also revealed three possible subgroups (Figs 2c and 3b). By performing the same analysis described above, we identified gene clusters similar to cluster CA1 containing 86 genes (cluster CA2) and cluster CB1 containing 234 genes (cluster CB2). Accordingly, patients with AD were divided into AD_HH,_ AD_LL_, and AD_HL_ groups based on the expression profiles of genes in clusters CA2 and CB2. The members of the AD_HH_, AD_LL_, and AD_HL_ groups were the same in both the N-A1 and N-A2 sets (S3a Fig in Supplementary File).

### Robustness of three AD subgroups

As in the following, the classification of AD patients into the AD_HH_, AD_HL_, and AD_LL_ was expectedly robust. Importantly, variations were observed among the AD_HH_, AD_HL_, and AD_LL_ individuals when different P^t^_NA_ and P^t^_AN_ values were employed to determine the genes in the N-At set. However, for small changes in the P^t^_NA_ and P^t^_AN_ values, less variation in individuals was observed among subgroups; for example, when using P^t^_NA_ =  0.06 and P^t^_AN_ =  0.1 instead of P^t^_NA_ =  0.08 and P^t^_AN_ =  0.1 to determine the N-At. Herein, the gene set named N-A3 was defined as that consisting of genes in the N-A_t_ set and maximum number of genes in N-A_D1_ set with low P-values that could separate patients with NCI from those with AD. Meanwhile, the gene set named N-A4 was defined as that consisting of genes in the N-A_t_ set and maximum number of genes in N-A_D2_ set with low P-values that could separate patients with NCI from those with AD. The classification of patients with AD into the AD_HH_, AD_LL_, and AD_HL_ groups was similar to that obtained for N-A1, N-A2, N-A3, and N-A4, where 24 of the 28 individuals belonged to the same subgroups (S3 Fig in Supplementary File). This suggested the robustness of our method in classifying the AD_HH_, AD_HL_, and AD_LL_ individuals.

In addition, the classification result of patients with AD into AD_HH_, AD_HL_, and AD_LL_ individuals seemed to be independent of the ages of individuals. For example, the numbers of individuals aged over and under 90 years in AD_HH_ were eight and two, those in AD_LL_ were six and four, and those in AD_HL_ were four and four, respectively (P-value =  0.4850).

### Common functional features of the N-A1 and N-A2 sets

In this subsection, we considered the common functional features of the N-A1 and N-A2 sets. The N-A1 set contained 1477 genes (1020 upregulated and 457 downregulated, AD), with 933 genes (683 upregulated and 250 downregulated) annotated as protein coding or noncoding RNA genes using Entrez [60] (Fig 3a). The N-A2 set contained 1424 genes (947 upregulated and 477 downregulated, AD), with 889 genes (619 upregulated and 270 downregulated) annotated as protein coding or noncoding RNA genes using Entrez [60] (Fig 3b). The upregulated genes in both the N-A1 and N-A2 set contained *AR*, *CBS*, *CLU*, *CYP19A1*, *DBH*, *GNA11*, *HLA-A*, *IQCK*, *MPO*, *MS4A4E*, *MX1*, *NLRP3*, *SDC2*, *TAP2*, and *ZFHX3* (S3d and S3e Tables), all of which have been reported to be AD risk-associated genes in recent studies [61,70–72]. Similarly, the downregulated genes in both the N-A1 and N-A2 set contained *COL25A1* and *TARDBP* (S3d and S3e Tables), which have also been reported to be AD risk-associated genes [61].

Next, we performed an enrichment analysis of all genes, including those with higher and lower expression levels in patients with AD for both the N-A1 and N-A2 sets (S3f–S3o Tables). We identified that genes with higher expression levels in patients of AD in both the N-A1 and N-A2 set were commonly enriched in GO terms for “forebrain dorsal/ventral pattern formation,” “mRNA 5′-splice site recognition,” and “negative regulation of NF-kappaB transcription factor activity” (P-value <  10^-3^ and FDR <  0.5) (S3g and S3j Tables). Notably, in the nervous system, NF-κB is known as one of the crucial components in the molecular switch that converts short- to long-term memory [73]. Thus, the upregulation of genes that suppress NF-κB expression is expected to exacerbate AD pathology.

Whereas, genes with lower expression levels in patients with AD in both the N-A1 and N-A2 set were enriched in GO terms for “negative regulation of intrinsic apoptotic signaling pathway in response to DNA damage by p53 class mediator,” “negative regulation of DNA damage response, signal transduction by p53 class mediator,” and “coronary vasculature morphogenesis” (P-value <  10^-3^ and FDR <  0.5) (S3h and S3k Tables). Here, the downregulation of genes that suppress apoptosis in response to DNA damage would also promote AD progression through the enhancement of cell death in the central nervous system [74,75].

### Functional features of gene sets classifying AD individuals into three subgroups

Now, we focused on the functional features of genes in the clusters CA1, CB1, CA2, and CB2 in the N-A1 and N-A2 sets that classify AD individuals into three subgroups by their expression levels. The clusters CA1, CB1, CA2, and CB2 contained 71, 215, 64, and 181 annotated genes, respectively (Fig 3). We identified *CLU*, *SDC2*, and *ZFHX3* in both the CA1 and CA2 clusters, whereas *DBH*, *GNA11*, *IQCK*, *MPO*, and *NLRP3* were identified in both the CB1 and CB2 clusters (S3d and S3e Tables), thereby suggesting that these AD risk genes are responsible for the differences in the features among the three AD subgroups [61,72].

Additionally, we performed enrichment analysis and found that genes in both the CA1 and CA2 clusters were enriched in GO terms for “negative regulation of carbohydrate metabolic process” and “neuron migration” (P-value <  10^-3^ and FDR <  0.5) (S3l and S3n Tables). Notably, the perturbed cerebral carbohydrate metabolism contributes to AD pathogenesis [76,77], whereas neuron migration ability contributes to neurogenesis in the brain [78,79]. These results suggested that the three AD subgroups may differ in these functional features.

Genes in both the CA1 and CA2 clusters were also enriched in GO terms for “response to xenobiotic stimulus,” “cellular response to lipid,” “negative regulation of small molecule metabolic process,” “lung development,” “cellular response to external stimulus,” and “positive regulation of epithelial cell differentiation” (P-value <  10^-3^ and FDR <  0.5) (S3l and S3n Tables). Meanwhile, genes in both the CB1 and CB2 clusters were enriched in GO terms for “mitotic spindle assembly checkpoint signaling” and “L-alpha-amino acid transmembrane transport” (P-value <  10^-3^ and FDR <  0.5) (S3m and S3o Tables).

### Epigenome data analysis supported the classification of patients with AD into three groups

Rush AD database also included data on the genome-wide distribution of epigenomic markers, H3K4me3, H3K27me3, and H3K27ac and CTCF binding site, for approximately half of all individuals ( S1a and S1c Tables). Thus, in this section, the classification of individuals based on epigenome features was performed. As mentioned in the following, epigenome features are also exhibited differently among AD_HL_, AD_LL_, and AD_HH_ individuals.

**Fig 4:**
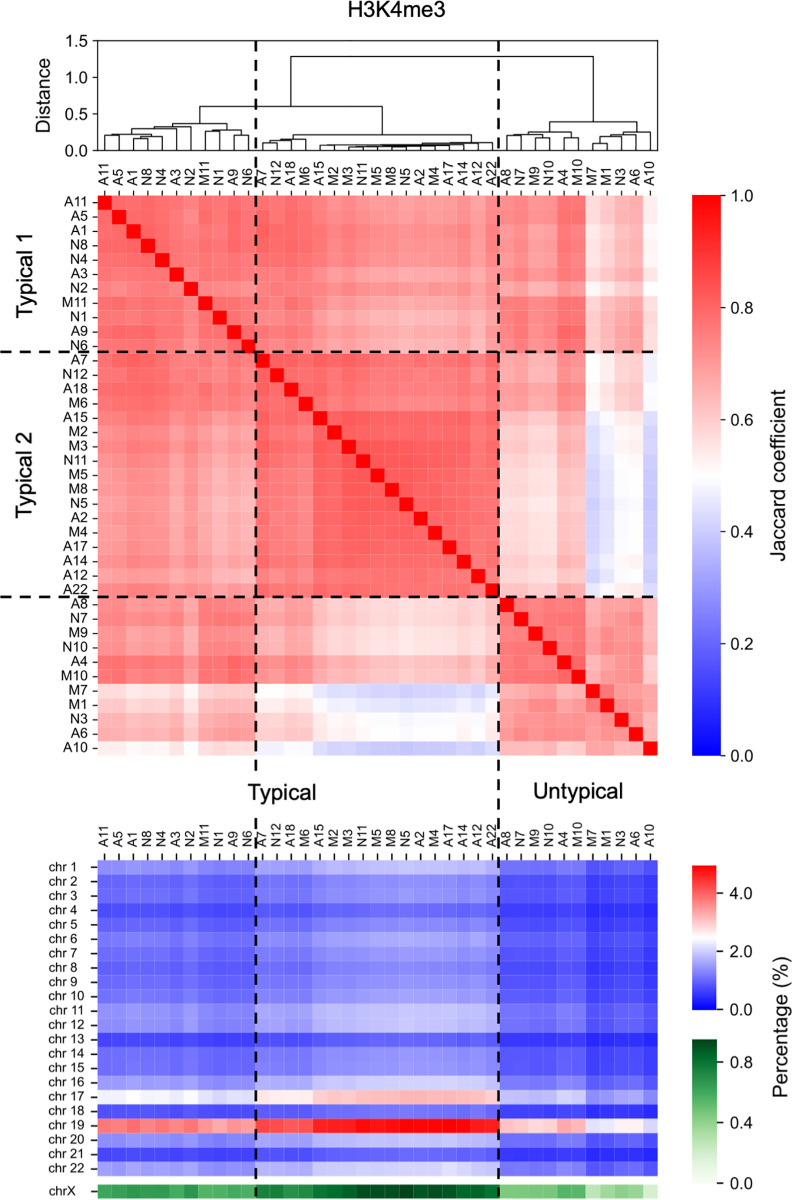
Jaccard coefficient of the genome-wide distribution of H3K4me3 among individuals. Individuals were divided into two groups at the first divergence of the dendrogram. The epigenetic state exhibited by individuals belonging to the group containing the larger number of individuals was regarded as the “typical” epigenetic state, whereas that exhibited by individuals belonging to the group containing the fewer individuals was regarded as the “untypical” epigenetic state. Further, individuals belonging to the “typical” group were divided into two groups, named Typical 1 and Typical 2, at the second divergence of the dendrogram. Dashed lines indicate the boundaries between groups.

**Fig 5:**
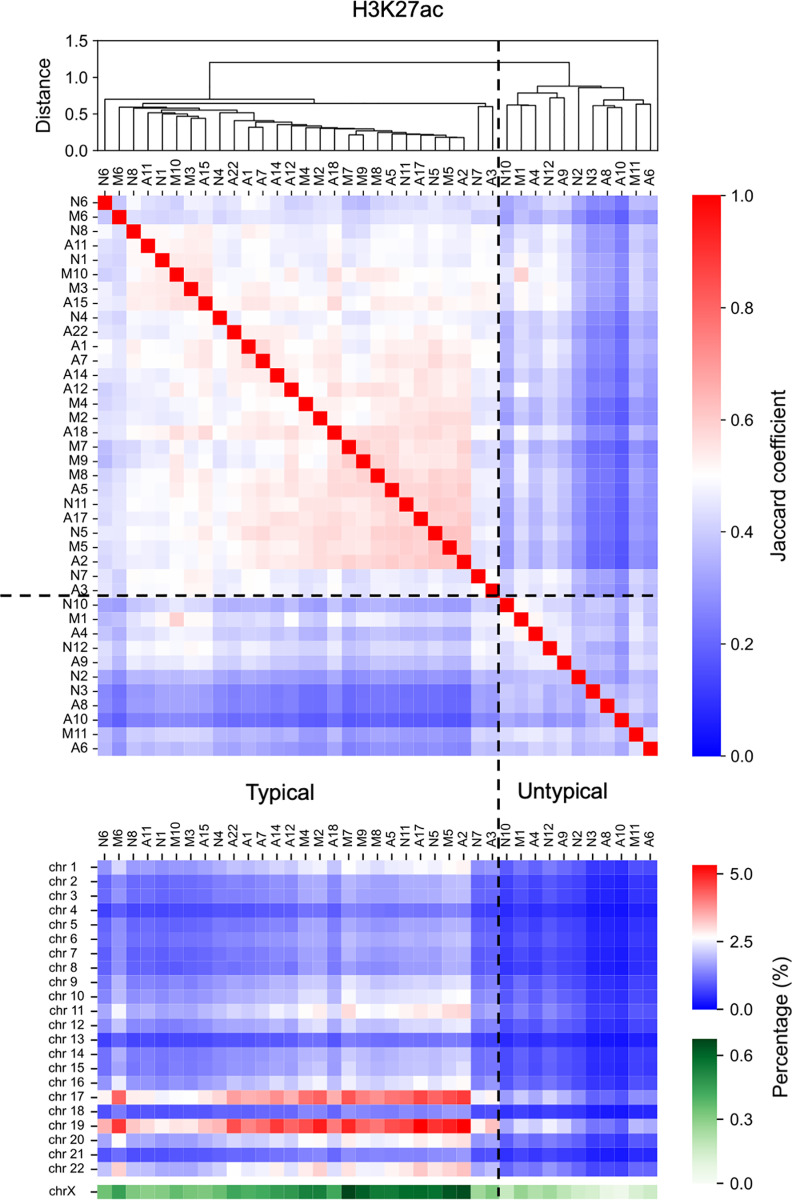
Jaccard coefficient of the genome-wide distribution of H3K27ac among individuals. Analysis was conducted similar to that described in Fig 4.

First, we evaluated the similarity of such epigenomic features among individuals using Jaccard coefficients of the genome-wide distribution of peak regions of epigenome status (Figs 4 and 5, S4 Table and S4 Fig in Supplementary File). According to hierarchical clustering using Jaccard coefficients, individuals were divided into two groups at the first divergence of the dendrogram: a majority and a minority group. Here, for the respective epigenomic markers, we assumed the epigenome status distribution of the majority group as “typical,” and another one was considered “untypical” (Figs 4 and 5, S4 Fig in Supplementary File). The Jaccard coefficients of peak regions of epigenome status among individuals suggested that more AD_HL_ individuals exhibited untypical patterns compared with those in other patients with AD (Figs 4 and 5, S4 Fig in Supplementary File, Table 2); in particular, the AD_HL_ group had a significantly larger number of patients with H3K4me3 modifications compared with the AD_HH_ and AD_HL_ groups (Table 2a). Similar features were obtained for H3K27ac, H3K27me3, and CTCF (Tables 2b–2d); however, these results were not significant. Moreover, AD_HL_ individuals tended to exhibit low epigenetic peak region density (Figs 4 and 5, S4 Fig in Supplementary File).

**Table 2:** Number of individuals exhibiting several genome-wide distributions of each epigenome marker. Number of individuals of AD_HH_, AD_LL_, and AD_HL_ exhibiting typical or untypical genome-wide distributions of H3K4me3 (a), H3K27ac (b), H3K27me3 (c), and CTCF (d). Number of individuals of AD_HH_, AD_LL_, and AD_HL_ exhibiting first type (Typical 1), second type (Typical 2) or untypical genome-wide distributions of H3K4me3 (e). Number of individuals of NCI, MCI, and AD exhibiting Typical 1, Typical 2 or untypical genome-wide distributions of H3K4me3 (f). Number of individuals exhibiting each genome-wide epigenome status in each substage or stage was obtained from **Fig 4** and **5**, and **S4 Fig** in Supplementary File. P-values obtained using the Fisher’s exact test were 0.04328 (a), 0.193439 (b), 0.193439 (c), 0.285294 (d), 0.042163, and 0.428061 (f).

(a)			
H3K4me3	AD_HH_	AD_LL_	AD_HL_
# of individuals with typical	6	4	3
# of individuals with untypical	0	0	4
(b)			
H3K27ac	AD_HH_	AD_LL_	AD_HL_
# of individuals with typical	5	4	3
# of individuals with untypical	1	0	4
(c)			
H3K27me3	AD_HH_	AD_LL_	AD_HL_
# of individuals with typical	5	4	3
# of individuals with untypical	1	0	4
(d)			
CTCF	AD_HH_	AD_LL_	AD_HL_
# of individuals with typical	6	3	4
# of individuals with untypical	0	1	3
(e)			
H3K4me3	AD_HH_	AD_LL_	AD_HL_
# of individuals with Typical 1	1	2	2
# of individuals with Typical 2	5	2	1
# of individuals with untypical	0	0	4
(f)			
H3K4me3	NCI	MCI	AD
# of individuals with Typical 1	5	1	5
# of individuals with Typical 2	3	6	8
# of individuals with untypical	3	4	4

Note that the optimal number of clusters for hierarchical clustering of individuals based on the Jaccard coefficients of the genome-wide distributions of H3K4me3 marker was obtained as three by the elbow method (S4c Fig in Supplementary File). Therefore, the following case was considered: individuals exhibiting typical H3K4me3 status were further divided into two groups (named Typical 1 and Typical 2) by the second branch, resulting in three clusters, including the untypical cluster (Fig 4). Based on such specific epigenome features, more AD_HH_ individuals belonged to the Typical 2 subgroup compared with other patients with AD (Table 2e). These facts suggest that the AD_HH_, AD_HL_, and ADLL individuals exhibited differences in the genome wide distribution of H3K4me3 modifications.

We also note that such significant differences in the genome wide distribution of H3K4me3 modifications were not obtained among individuals in NCI, MCI, and AD (Table 2f).

### Relationships between transcriptome and H3K4me3 variations among AD subgroups

Results of both transcriptome data analysis and epigenome data analysis, particularly the analysis of H3K4me3, suggested that AD stage was divided into three substages, AD_HH_, AD_HL_, and AD_LL_. Subsequently, the relationship between the differences in the distribution of H3K4me3 and differences in gene expression levels among the three AD subgroups was focused. Notably, AD individuals were divided into three substages according to the expression levels of genes in CA1 and CB1 in the N-A1 set or CA2 and CB2 in the N-A2 set. Thus, for the common genes in CA1 and CA2 and those in CB1 and CB2, we calculated the correlation between the expression level of each gene and H3K4me3 marker density in the promoter region of that gene for AD individuals.

**Fig. 6:**
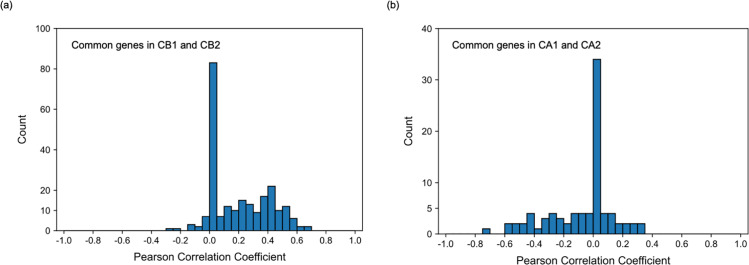
Histograms of the correlation coefficient between expression levels of genes and epigenome marker densities on promoters of these genes that divided AD individuals into three AD substages. Histograms of the correlation coefficient for common genes in CB1 and CB2 (Fig. 3) (a) and that for common genes in CA1 and CA2 (Fig. 3) (b). Since there are genes whose promoters did not have H3K4me3 marks, a peak appears at correlation =  0. However, in (a), a high peak was seen even in areas with high correlation. (See S4e Table for correlation coefficient of each gene.).

Resultingly, many of the common genes in CB1 and CB2 tended to be upregulated with increasing H3K4me3 density in their promoters (Fig. 6a and S4e Table). This suggests that the distribution of H3K4me3 contributes to the appearance of three substages of AD through the regulation of the expression levels of some genes. In contrast, many of the common genes in CA1 and CA2 did not show such a tendency (Fig. 6b and S4e Table). Therefore, it was suggested that the effect of H3K4me3 on the multistate nature of AD is partial.

### No effect of *APOE* gene polymorphisms in classifying individuals

In this subsection, we examined the relationships between the *APOE* genotype and the subgroup to which individuals belong. The *APOE* genotype of each individual was identified using the RNA-seq read data of individuals in the Rush AD database. Accordingly, no individuals were found to simultaneously possess the ε2 and ε4 alleles. Therefore, individuals were classified in three categories: APOE2 carrier, possessing one or two ε2 alleles; APOE3 carrier, possessing two ε3 alleles; and APOE4 carrier, possessing one or two ε4 alleles. Note that all APOE2 carriers (12 individuals) were identified as hetero APOE2 carriers with only one ε2 allele, and all APOE4 carriers except an individual (15 individuals) were identified as hetero APOE4 carriers with only one ε4 allele where only N9 individual was identified as a homo APOE4 carrier with two ε4 alleles.

We obtained the number of APOE2, APOE3, and APOE4 carriers did not seem to correlate with the clinical diagnostic stage of individuals (Table 3a, S1a Table). In addition, the number of individuals also seemed to be independent of the two MCI (MCI_Major_ and MCI_NCI_AD_) and three AD (AD_HH_, AD_HL_, and AD_LL_) substages (Tables 3b and 3c, S1a Table).

**Table 3:** Number of APOE2, APOE3, and APOE4 carriers in each clinical diagnostic stage (a), in the two MCI substages (b), and in the three AD substages (c). The genotype of each individual is given in S1a Table. The P-values obtained using the Fisher’s exact test were 0.8841 (a), 0.08148 (b), and 0.4379 (c).

(a)				
Clinical Diagnostic Stage	NCI	MCI		AD
# of APOE2 carriers	5	3		4
# of APOE3 carriers	16	20		18
# of APOE4 carriers	4	6		6
(b)				
MCI substages	MCI_Major_		MCI_NCI_AD_	
# of APOE2 carriers	1		2	
# of APOE3 carriers	18		2	
# of APOE4 carriers	5		1	
(c)				
AD substages	AD_HH_	AD_LL_		AD_HL_
# of APOE2 carriers	3	1		0
# of APOE3 carriers	6	7		5
# of APOE4 carriers	1	2		3

### Adjacency network among substages

To clarify the relationships among disease substages and the pathways of disease progression in these substages, the adjacency network among substages was estimated. The data on the expression levels of genes in the M-NA set revealed differences between patients with typical MCI, NCI, and AD, whereas those in the N-A1 or N-A2 sets revealed differences between patients with NCI and AD. Thus, we explored the adjacency networks among substages using M-NA and N-A1 sets, and M-NA and N-A2 sets by performing the partition-based graph abstraction (PAGA) method [59]. Using PAGA, we generated a graph of substages in which the edge weights represented connection confidence. Note that connection confidence between NCI and AD substages often involved finite values (S5 Table); however, we expected that at least one of the MCI stages is undergone during the progression from NCI to AD because AD progresses gradually over 10 years (in some cases over 20 years) after onset [80] Therefore, we assumed that only connections with confidence values sufficiently larger than the maximum confidence values of the connection between NCI and AD substages (MD_N_A_) were effective for the adjacency network among substages (S5 Fig in Supplementary File). Subsequently, we used connections with confidence values larger than twice that of MD_N_A_ to construct the adjacency network (S5 Table).

**Fig 7:**
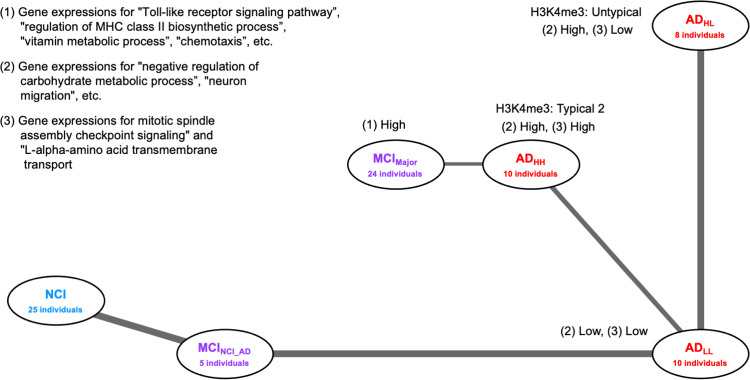
Adjacency network among substages. The network constructed using common connections obtained from both the network constructed using the M-NA and N-A1 sets (S5a Fig) and that constructed using the M-NA and N-A2 sets (S5b Fig). The width of each edge indicates the connection confidence between each pair of connected nodes.

Changes in gene expression patterns that characterize disease states are considered to occur gradually as the disease progresses. Therefore, we observed that disease appeared to progress from NCI to AD along the previously obtained adjacency network. Note that the adjacency network obtained using the M-NA and N-A1 sets (S5a Fig in Supplementary File) and that constructed using the M-NA and N-A2 sets (S5b Fig in Supplementary File) exhibited a slightly different structure because of differences in the contained genes in these sets. Thus, we focused on the network constructed using common connections obtained from both networks constructed using the M-NA and N-A1 sets (S5a Fig in Supplementary File) and M-NA and N-A2 sets (S5b Fig in Supplementary File) (Fig 7). We did not obtain any connections from NCI to MCI_Major_, the major group of MCI individuals, in any of the networks (Fig 7, S5a and S5b Figs in Supplementary File), which seemed unnatural for AD progression. Thus, we performed more detailed subgroup classifications of patients with NCI, MCI, and AD to reveal the relationship between NCI and MCI_Major_ individuals.

### Detailed classification of patients with MCI by comparing between patients with NCI and those with MCI and between patients with MCI and those with AD

In this subsection, we suggested that MCI patients might be classified into four more detailed subgroups, as in the following.

**Fig 8:**
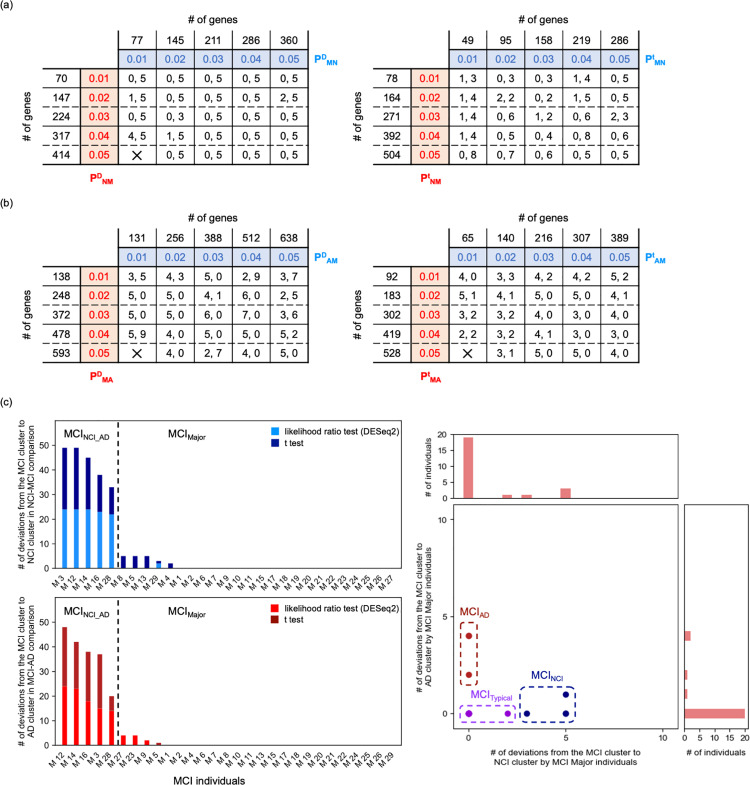
Detailed classification of patients with MCI. Number of patients with NCI who belong to the MCI cluster (left value) and that of patients with MCI who belong to the NCI cluster (right value) as a function of P^D^_NM_ and P^D^_MN_ (a, left), and as a function of P^t^_NM_ and P^t^_MN_ (a, right). Number of patients with MCI who belong to the AD cluster (left value) and that of patients with AD who belong to the MCI cluster (right value) as a function of P^D^_MA_ and P^D^_AM_ (b, left), and as a function of P^t^_MA_ and P^t^_AM_ (b, right). Number of deviations of each patient with MCI from the MCI to the NCI (c, upper left) and AD (c, lower left) cluster. Broken lines indicate boundaries between MCI_NCI_AD_ and MCI_Major_ individuals. Scatter plot of MCI_Major_ individuals as a function of the number of times that each individual belonged to the NCI and AD cluster (c, right). Using k-means clustering (S6c Fig), MCI_Major_ individuals were divided into MCI_Typical_, MCI_NCI_, and MCI_AD_ groups.

To classify patients with MCI more comprehensively, we conducted comparisons between patients with NCI and those with MCI and between patients with MCI and those with AD. First, we performed hierarchical clustering of individuals using the genes satisfying the following criteria: the mean expression level (TPM) was higher in the MCI group than that in the NCI group with smaller P-values in the likelihood ratio test than the P^D^_MN_ threshold or the mean expression level (TPM) was lower in the MCI group than that in the NCI group, with smaller P-values in the likelihood ratio test than the P^D^_NM_ threshold. We performed cluster analysis using each gene set within the range P^D^_NM_ ≤  0.05 and P^D^_MN_ ≤  0.05. However, we did not identify any cases in which patients with NCI were completely separated from those with MCI (Fig 8a, S6a Fig in Supplementary File). However, clusters with significantly more patients with NCI (NCI clusters) and MCI clusters were formed within all cases of P^D^_NM_ and P^D^_MN_ except in the case of P^D^_NM_ =  0.05 and P^D^_MN_ =  0.01 (Fig 8a).

When we used two-tailed *t*-tests instead of the likelihood ratio test to determine the P-values, our results were qualitatively the same. P^t^_MN_ and P^t^_NM_ were defined instead of P^D^_MN_ and P^D^_NM_, respectively. Analysis using each gene set within the ranges P^t^_MN_ ≤  0.05 and P^t^_NM_ ≤  0.05 revealed no cases in which patients with NCI were completely separated from those with MCI (Fig 8a, S6a Fig in Supplementary File). However, NCI and MCI clusters were formed within all cases of P^t^_NM_ and P^t^_MN_ (Fig 8a).

Next, we performed the same analyses for patients with MCI and those with AD. In this case, P^D^_MA_, P^D^_AM_, P^t^_MA_, and P^t^_AM_ were given as values corresponding to P^D^_NM_, P^D^_MN_, P^t^_NM_, and P^t^_MN_, respectively. Similar to the above results, we did not identify any cases in which patients with MCI were completely separated from those with AD (Fig 8b, S6b Fig in Supplementary File). However, MCI and AD clusters were formed within all cases of P^D^_MA_, P^D^_AM_, P^t^_MA_, and P^t^_AM_ except in the case of P^D^_MA_ =  0.05 and P^D^_AM_ =  0.01 and that of P^t^_MA_ =  0.05 and P^t^_AM_ =  0.01 (Fig 8b).

In this study, variations were observed in patients with MCI who deviated from the MCI cluster depending on P^D^_NM_, P^D^_MN_, P^t^_NM_, P^t^_MN_, P^D^_MA_, P^D^_AM_, P^t^_MA_, and P^t^_AM_ values. For each patient with MCI, we counted the number of times they belonged to the NCI cluster upon changing the P^D^_NM_, P^D^_MN_, P^t^_NM_, and P^t^_MN_ values, and those belonging to the AD cluster upon changing the P^D^_MA_, P^D^_AM_, P^t^_MA_, and P^t^_AM_ values (Fig 8c). Consequently, individuals M3, M4, M5, M8, M12, M13, M14, M16, M28, and M29 belonged to the NCI cluster more than once, whereas individuals M3, M5, M9, M12, M14, M16, M23, M27, and M28 belonged to the AD cluster more than once.

Notably, individuals M3, M12, M14, M16, and M28, (same as those in MCI_NCI_AD_), clearly exhibited a greater number of deviations from patients with NCI and AD than MCI_Major_ individuals (Fig 8c). To classify MCI_Major_ individuals in more detail, we performed k-means clustering using the number of times each individual belonged to the NCI cluster and that belonging to the AD cluster (Fig 8c, S6c Fig in Supplementary File). As a result, MCI_Major_ individuals were divided into three subtypes: MCI_Typical_ individuals (17 individuals) who rarely or never belonged to other clusters; MCI_NCI_ individuals (4 individuals) who may belong to NCI clusters only; and MCI_AD_ individuals (3 individuals) who may belong to AD clusters only (S1a Table). Thus, MCI was divided into four substages: MCI_Typical_, MCI_NCI_, MCI_AD_, and MCI_NCI_AD_.

### Detailed classification of patients with NCI by comparing them with patients with MCI

In this subsection, we suggested that NCI patients might be classified into two more detailed subgroups, as in the following.

**Fig 9:**
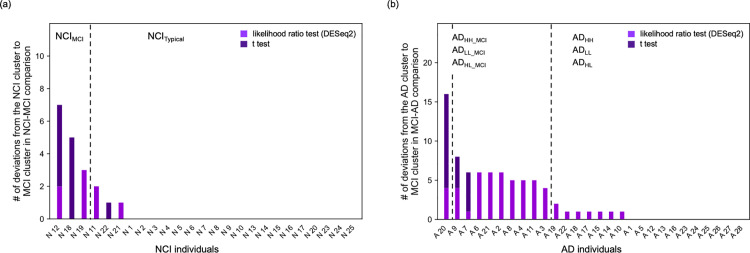
Detailed classification of patients with NCI or AD. Number of deviations of each patient with NCI from the NCI to the MCI cluster (a), and that of each patient with AD from the AD to the MCI cluster (b). Broken lines indicate boundaries among clusters of individuals obtained via k-means clustering (S7a and S7b Figs in Supplementary File).

Similar analysis to that performed in the previous subsection showed that individuals N11, N12, N18, N19, N21, and N22 belonged to the MCI cluster more than once when the P^D^_NM_, P^D^_MN_, P^t^_NM_, and P^t^_MN_ values were changed (Fig 9a). After k-means clustering of patients with NCI using the number of times each individual belonged to the MCI cluster, patients with NCI were divided into two clusters: one comprising N11, N21, and N22, and another comprising those who never belonged to the MCI cluster (Fig 9a, S7a Fig in Supplementary File). Therefore, these individuals were classified as typical patients with NCI belonging to the NCI cluster, named NCI_Typical_ individuals, who were assumed to be in the NCI_Typical_ substage. Individuals N12, N18, and N19 were classified as patients with NCI close to MCI, named NCI_MCI_ individuals, who were assumed to be in the NCI_MCI_ substage (S1a Table).

### Detailed classification of patients with AD by comparing them with patients with MCI

In this subsection, we suggested that AD patients might be classified into six more detailed subgroups, as in the following.

By the similar analysis to that in the previous subsection, 17 patients with AD belonged to the MCI cluster more than once, when the P^D^_MA_, P^D^_AM_, P^t^_MA_, and P^t^_AM_ values were changed (Fig 9b). Using k-means clustering of patients with AD based on the number of times each individual belonged to the MCI cluster, patients with AD were divided into three clusters, with individuals A10, A14, A15, A17, A18, A19, A22, and other patients with AD who never belonged to the MCI cluster being included in the same cluster (Fig 9b, S7b Fig in Supplementary File). Therefore, these individuals were expected to be in one of the typical AD substages: AD_HH_, AD_HL_, or AD_LL_ (S1a Table). In contrast, individuals A2, A3, A4, A6, A7, A8, A9, A11, A20, and A21 were assumed to be at one of the AD substages close to MCI and were named AD_HH_MCI_, AD_HL_MCI_, or AD_LL_MCI_ (S1a Table).

### Adjacency network among substages obtained after detailed classifications of individuals suggests multiple disease progression pathways from NCI to AD

The adjacency network among substages after detailed classifications was evaluated to clarify the relationships among detailed substages and the pathways of disease progression in these substages.

**Fig 10:**
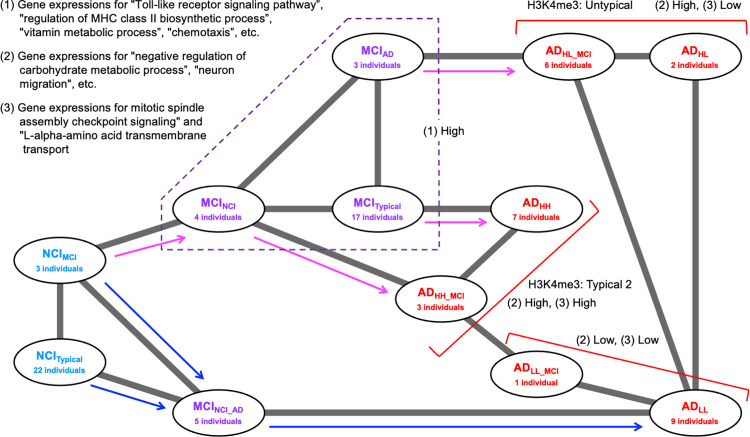
Adjacency networks among more detailed substages. A network containing common connections from both networks obtained using the data in the M-NA and N-A1 sets (S8a Fig in Supplementary File) and that in the M-NA and N-A2 sets (S8b Fig in Supplementary File). The width of each edge indicates the connection confidence between each pair of connected nodes. Arrows indicated the inferred direction of disease progression.

Detailed classifications of individuals suggested that patients with NCI were classified into NCI_Typical_ and NCI_MCI,_ patients with MCI were classified into MCI_Typical_, MCI_NCI_, MCI_AD_, and MCI_NCI_AD,_ and patients with AD were classified into AD_HH_, AD_HH_MCI_, AD_HL_, AD_HL_MCI_, AD_LL_, and AD_LL_MCI_. We constructed adjacency networks among these 12 substages using gene expression data from the M-NA and N-A1 sets (S8a Fig in Supplementary File, S6a Table) as well as from the M-NA and N-A2 sets (S8b Fig in Supplementary File, S6b Table) as previously described. Subsequently, we focused on the adjacency network that consisted of common connections between the network from the M-NA and N-A1 sets, and that from the M-NA and N-A2 sets (Fig 10). We identified more than four progression paths from NCI to AD that passed through different substages, as follows: I) from NCI (NCI_Typical_ or NCI_MCI_) to AD_LL_ through MCI_NCI_AD_, II) from NCI to MCI_NCI_ and from MCI_NCI_ to AD_LL_ through AD_HH_MCI_ and AD_LL_MCI_, respectively, III) from NCI to MCI_NCI_ and from MCI_NCI_ to AD_HH_ through MCI_Typical_ or AD_HH_MCI_, and IV) from NCI to AD_HL_ or AD_LL_ via MCI_NCI_, MCI_AD_, or AD_HL_MCI_. This suggested that at least four disease progression pathways are active in the three classified AD substages.

### Comparisons with the findings of recent studies

Many recent studies have performed transcriptome analyses and identified various significant differentially expressed genes (significant DEGs) between healthy (control) individuals and patients with AD, which they term“d “AD risk genes” [61,70–72]. The cluster analysis of patients with NCI, MCI, and AD and that of patients with NCI and AD was performed using these AD risk genes (S9 Fig in Supplementary File, S7 Table). However, these results differed from the individual clustering results in Figs 1–3. Notably, most of the AD risk gene data used here were obtained from research on the hippocampus [61,70–72], whereas the data analyzed in this study were obtained from the prefrontal cortex. A recent study showed that gene expression patterns differ between the hippocampus and prefrontal cortex [9]. Hence, the observed inconsistencies were attributed to these differences.

From the dataset analyzed in the previous sections, no significant DEGs were detected between patients with NCI and those with AD, whereas only nine significant DEGs were identified among patients with NCI, MCI, and AD (S1b and S8 Tables). Herein, significant DEGs were evaluated via the likelihood test using DEseq2 with FDR <  0.01. Notably, the abovementioned study reported that not many significant DEGs were yielded in areas belonging to the prefrontal cortex [9]. These facts supported the consistency between the results of the present and those of recent studies.

## Discussion

In this study, the analysis of transcriptome data from women diagnosed with NCI, MCI, and AD, obtained from the Rush AD database, suggested that NCI, MCI, and AD can be classified into two, four, and six substages, respectively.

Differences among AD substages were identified by transcriptome data and epigenomic features, particularly the genome-wide distribution of H3K4me3. However, the *APOE* gene polymorphisms of individuals appeared to not correlate with the disease substage. In addition, some of the genes and gene sets that were suggested in recent studies to be AD-associated contributed to the characterization of each AD substage in the present study. Second, an inference of adjacency networks among the different substages using transcriptome data suggested the existence of four typical NCI →  AD disease progression pathways from different MCI to different AD substages. These facts suggest that the pathological progression pathways of AD are multiple and heterogeneous, with various typical forms of deterioration.

These findings on disease progression features will facilitate further advancements in AD research. For example, identifying biomarkers [81–83] that characterize each substage will enable a more precise diagnosis of AD based on these findings. Further, the development of medical interventions and drug discovery/administration strategies suited to the gene expression and epigenetic status of each substage, as well as predicted disease progression, is expected to lead to the development of more extensive prevention, patient care, and treatment.

Recent studies using hippocampal and frontal lobe transcriptomes have also suggested the existence of multiple AD subtypes [9]. However, the relationship between multiple states, such as their connection in a series on the same disease progression path or on different paths, has not been clarified. Furthermore, these studies did not focus on the diversity of NCI and MCI. In contrast, in this study, both patients with NCI and MCI were divided into multiple subgroups at different substages based on their respective gene expression characteristics. In addition, estimating the connectivity among substages clearly revealed the existence of multiple pathological pathways of disease progression.

This study analyzed data from the dorsolateral prefrontal cortex obtained from the Rush AD database, rather than the hippocampus, which has been the focus of many previous studies. The reason for analyzing this dataset in this study was that it included not only transcriptome data but also a variety of data on the genome-wide epigenome status such as the distribution of histone modifications (H3K4me3, H3K27me3, H3K27ac) and CTCF binding sites (S1a and S1c Tables). Recently, some relationships between epigenome states of brain cells and AD pathology were suggested. The aberrant localization of H3K4me3 in the hippocampus was found from an early stage of AD [84]. Additionally, the associations between the changes in transcription activities of some synaptic genes and changes in CTCF binding and H3K27ac states near these genes were shown to be associated with changes in pathological conditions [85]. Interestingly, the analysis of epigenome data in the present study suggests that the genome-wide distribution of the H3K4me3 marker in dorsolateral prefrontal cortex cells did not show clear differences among NCI, MCI, and AD individuals. However, the distribution of the H3K4me3 marker differed among individuals belonging to three different AD substages. Additionally, the differences in the expression levels of some genes that characterized the differences among three AD substages were positively associated with the differences in the density of the H3K4me3 marker in the promoter region of such genes. These facts suggest that H3K4me3 in the brain was associated with not only AD progression, as observed in the hippocampus, but also differentiation of AD pathology.

Notably, many recent studies have performed transcriptome analyses to identify significant DEGs between healthy individuals and patients with AD [61,70–72]. However, by focusing on the expression patterns of more widespread candidate genes rather than the significant DEGs, we identified different characteristics appearing in the dorsolateral prefrontal cortex between healthy individuals and patients with AD and that disease substages occur in response to changes in the disease state.

In addition, many studies have focused on gene expression patterns in the hippocampus [9,61,70–72]. One such previous research suggested the existence of multiple states in the hippocampus of patients with AD based on differences in the gene expression profiles [9]. However, as previously mentioned, the gene expression patterns differ between the hippocampus and prefrontal cortex, and these differences exhibit further variations between healthy individuals and patients with AD [9,61,70–72]. Hence, different results would be obtained even from the same analysis (See “Comparisons with the findings of recent studies” subsection in the Results section.). Importantly, the hippocampal data analyzed in previous studies [9] can be analyzed in the same way as in this study; connections between substages can be extracted, and pathological routes can be estimated, all of which are crucial issues that need to be addressed in the future.

We obtained the results that the *APOE* gene polymorphisms of individuals did not correlate with the disease substages. This seems to contradict the results of large-scale surveys previously reported [86,87]. The reasons for this were thought to be as follows. First, the number of subjects in the database used in this study was smaller than two orders of magnitude than in previous studies of *APOE* polymorphisms. Second, all but one APOE4 carrier were heterozygous carriers, so the influence of *APOE4* may not be as strong in everyone.

Therefore, we do not deny the possibility that the relationship between NCI, MCI, and AD may approach that reported previously and that some differences may occur between AD subgroups if we investigate more individuals.

However, compared to epigenetic properties such as gene expression and epigenetic state, which are sufficiently affected by the current number of individuals, the impact on the pathology might be small.

The present study has some limitations. We only classified female individuals as we used transcriptome and epigenomic data of female patients from the Rush AD database. However, the insights of this study are expected to be useful not only for female patients but also for understanding the rapid progression of symptoms in male patients. To validate this expectation, the dataset from male individuals should also be analyzed. In this dataset, however, the number of female patients in the NCI, MCI, and AD groups was 25, 29, and 28, respectively, whereas that of men was 14, 4, and 7, respectively, that is, three times smaller than that of female individuals (seven and four times smaller than those of female patients with MCI and AD). Moreover, the number of male patients with MCI and AD in the database (four and seven) was smaller than that of individuals in each of the three AD groups obtained for female individuals. With such restricted data, performing similar analyses on male individuals or sex-independent analyses was impossible.

In addition, this study could not consider whether there are differences in which substages males and females are more likely to develop, or whether there may be substages specific to males and females. It was also unable to consider how differences in the activity of genes such as PAPP-A and KDM6A, which are particularly active in females and have recently been suggested to be associated with AD [41–43], affect the pathology between males and females. Furthermore, it has been reported that the progression rate is faster in men [42,43], and it was predicted that this is due to the expression of the KDM6A gene, which is in the region that is not inactivated on both X chromosome pairs in women. However, this study could not clarify whether such genes have any effect on the progression rate or other pathology. Obtaining more data on male patients is one of the essential challenges that must be overcome in the future for performing analyses of such issues and eliminating potential sex-related bias.

Since there were only a few NCI or MCI individuals other than NCI_typical_ or MCI_typical_, there were very few NCI or MCI individuals other than NCI_typical_ or MCI_typical_ with epigenomic data (See S1a Table). Therefore, useful statistical analysis and interpretation are difficult, and we were not able to have a clear discussion in this paper. We would like to consider this when more data is obtained in the future.

The likelihood ratio test using DESeq2 and *t*-test were used to evaluate the differences in RNA-seq data between different groups when searching for gene sets to characterize and classify individuals. The *t*-test is generally better for detecting differences and was expected to identify more candidate genes in a gene set than the likelihood ratio test or other nonparametric testing methods. However, the *t*-test produces more false positives than other testing methods when data deviate from a Gaussian distribution. The gene sets used in this study were expected to contain few false-positive genes because they were chosen under imposing the additional condition that healthy individuals could be clearly distinguished from patients through cluster analysis. However, inappropriate genes may have still been retained. For more precise consideration, verification is necessary using various nonparametric tests.

In addition to the transcriptome of each individual, the database targeted for the present analysis also contained various epigenome data; however, other information on each individual, such as postmortem interval, brain PH, blood tests, protein biomarker levels, distributions of Aβ plaques and abnormal tau aggregations in the brain, and medical history was not included. Therefore, although this study has yielded some unprecedented findings, the validation and characterization of each substage through comparisons with other physiological data could not be included. For example, the relationship between the multiple pathologies and progression of AD obtained in this study and the numerous manners of spreading abnormal tau aggregations in the brain reported in previous studies [44] could not be considered. However, such analysis may be achieved by combining meta-analysis with databases focusing on other feature groups.

## Supporting information

S1 Table Detailed information of analyzed data.
ID of individuals in this study, ID in the ENCODE database, diagnostic stage, inferred substage, inferred detailed substage, first, second, and third quantiles of MOR, availability of epigenome data, *APOE* genotype estimated using RNA-seq data (a). The results of the likelihood test by DEseq2, ANOVA, and the two-tailed *t*-test of expression levels of genes (b). Total number of reads from ChIP-seq data for H3K27ac, H3K27me3, H3K4me3, and CTCF mapped to the human genome (GRCh38) (c).
(XLSX)

S2 Table Detail features of M-NA set.
Gene list of M-NA set where the order of genes was the same as that in Fig 1b (a). Detailed results of the enrichment analysis were obtained using all genes in the M-NA set (b), those with higher expression levels in MCI_Major_ individuals (c), and AD risk genes with lower expression levels in MCI_Major_ individuals (d).
(XLSX)

S3 Table Detail features of N-AA and N-A2 set.
List of genes in the N-A_D1_ set (a), that in the N-A_D2_ set (b), that in the N-A_t_ set (c), that in the N-A1 set (d), and that in the N-A2 set (e). The order of genes in (d) and (e) are the same as that in Fig 3a and 3b, respectively. Genes in clusters CA1, CB1, CA2, and CB2 are also indicated. The P-values of ANOVA with the result of the Benjamini–Hochberg procedure (FDR <  0.1) of the expression level of each gene when the NCI stage is divided into three or four subgroups, and that of each gene among the three subgroups of AD, are also shown. Detailed results of the enrichment analysis were obtained using the genes in the N-A1 set (f), genes in the N-A1 set with higher expression levels in AD than in NCI (g), genes in the N-A1 set with lower expression levels in AD than in NCI (h), genes in the N-A2 set (i), genes in the N-A2 set with higher expression levels in AD than in NCI (j), and genes in the N-A2 set with lower expression levels in AD than in NCI (k). Detailed results of the enrichment analysis obtained using genes in clusters CA1 (l), CB1 (m), CA2 (n), and CB2 (o).
(XLSX)

S4 Table Detailed results of analysis of epigenome status and relationships between epigenome and transcriptome variations.
Values of Jaccard coefficients of genome wide maker peak regions among individuals (Upper) and density of marker for each chromosome of each individual (Lower) for H3K4me3 (a), H3K27ac (b), H3K27me3 (c), and CTCF (d). Correlation coefficient between expression levels of genes and H3K4me3 marker densities on promoters of these genes; correlation coefficient for common genes in CB1 and CB2 (Fig. 3), and that for common genes in CA1 and CA2 (Fig. 3) (e).
(XLSX)

S5 Table Matrix of confidence regarding the connections of adjacency networks among substages.
Matrix of confidence regarding the connections among patients with NCI, MCI_Major_, MCI_NCI_AD_, AD_HH_, AD_HL_, and AD_LL_ individuals estimated from the expression levels of the M-NA and N-A1 sets (a), and M-NA and N-A2 sets (b).
(XLSX)

S6 Table Matrix of confidence regarding the connections of adjacency networks among more detailed substages.
Matrix of confidence regarding the connections among the NCI_Typical_, NCI_MCI_, MCI_Typical,_ MCI_NCI_, MCI_AD_, MCI_NCI_AD_, AD_HH_MCI_, AD_HL_MCI_, AD_LL_MCI_, AD_HH_, AD_HL_, and AD_LL_ individuals estimated from the expression levels of the M-NA and N-A1 sets (a), and M-NA and N-A2 sets (b).
(XLSX)

S7 Table List of AD risk genes. AD risk genes previously reported in the literature (Hu et al. 2017; Xiang et al. 2018; Rahman et al. 2019; Yang et al. 2022) where the order of genes was the same as that in S9 Fig. Many of these AD risk genes were obtained from studies using the hippocampus.
(XLSX)

S8 Table Significant DEGs among patients with NCI, MCI, and AD.
(XLSX)

S1 File S1 Fig. Dendrograms of the hierarchical clustering among the investigated individuals.
Dendrograms of the hierarchical clustering among individuals when P^D^_NMA_ =  0.01 (a), 0.02 (b), 0.03 (c), 0.04 (d), and 0.05 (e), and when P^A^_NMA_ =  0.01 (f), 0.02 (g), 0.03 (h), 0.04 (i), and 0.05 (j). **S2 Fig. Dendrograms of hierarchical clustering among patients with NCI and AD.** Dendrograms of the hierarchical clustering among patients with NCI and AD obtained using the N-AD1 (a), N-AD2 (b), and N-At (c) sets. **S3 Fig. Validation of robustness of results of transcriptome data analysis.** Dependencies of belonging to each subgroup of patients with AD based on the P^D^_NA_, P^D^_AN_, P^t^_NA,_ and P^t^_AN_ values (a). Dendrograms of the hierarchical clustering among patients with NCI and AD obtained using the N-A3 (b, upper panel) and N-A4 (b, lower panel) sets. **S4 Fig. Results of epigenome data analysis.** Jaccard coefficient of the genome-wide distributions of H3K27me3 (a), and CTCF (b) among individuals. Individuals were divided into two groups at the first divergence of the dendrogram. The epigenetic state exhibited by individuals belonging to the group containing the larger number of individuals was regarded as the “typical” epigenetic state, whereas that exhibited by individuals belonging to the group containing the fewer individuals was regarded as the “untypical” epigenetic state. Dashed lines indicate the boundaries between groups. The sum of squared errors (SSE) as a function of the number of clusters for the hierarchical clustering of individuals based on the Jaccard coefficient of the genome-wide distributions of H3K4me3 (see Fig. 4) (c). This plot shows the inferred number of clusters =  3. **S5 Fig. Adjacency networks among substages.** Adjacency network obtained using the M-NA and N-A1 sets (a) and that constructed using the M-NA and N-A2 sets (b). **S6 Fig. Results of hierarchal clustering of patients with NCI and MCI and that of patients with MCI and AD.** Examples of the hierarchal clustering of patients with NCI and MCI (a) and patients with MCI and AD (b). The sum of squared errors (SSE) as a function of the number of clusters for the k-means clustering of MCI_Major_ individuals using the number of times each individual belonged to the NCI or AD clusters (c). This plot shows the inferred number of clusters =  3. **S7 Fig. Results of elbow method to evaluate cluster number.** The sum of squared errors (SSE) as a function of the number of clusters for k-means clustering of patients with NCI using the number of times each individual belonged to the MCI cluster (a), and that of patients with AD using the number of times each individual belonged to the MCI cluster (b). The number of clusters =  2 was inferred in (a), while the number of clusters =  3 was inferred in (b). **S8 Fig. Adjacency networks among more detailed substages.** Adjacency network obtained using the M-NA and N-A1 sets (a) and that constructed using the M-NA and N-A2 sets (b). **S9 Fig. Results of the cluster analysis using AD risk genes reported by recent literatures.** Results of the cluster analysis of patients with NCI, MCI and AD (a) and that of patients with NCI and AD (b) using AD risk genes (S7 Table) previously reported in the literature (Hu et al. 2017; Xiang et al. 2018; Rahman et al. 2019; Yang et al. 2022). Many of these AD risk genes were obtained from studies using the hippocampus. The gene expression patterns of the hippocampus generally differs from that of the prefrontal cortex examined in this study. Thus, the results obtained in this study were quite different from those from previous studies.
(DOCX)
