# Supplementary material for: Potential multiple disease progression pathways in female patients with Alzheimer’s disease inferred from transcriptome and epigenome data of the dorsolateral prefrontal cortex
Source: PLoS One. 2025 Mar 18;20(3):e0313733. doi: 10.1371/journal.pone.0313733 (PMC11918443; doi:10.1371/journal.pone.0313733)
Supplement: S1 File — Dendrograms of the hierarchical clustering among individuals when PDNMA = 0.01 (a), 0.02 (b), 0.03 (c), 0.04 (d), and 0.05 (e), and when PANMA = 0.01 (f), 0.02 (g), 0.03 (h), 0.04 (i), and 0.05 (j). S2 Fig. Dendrograms of hierarchical clustering among patients with NCI and AD. Dendrograms of the hierarchical clustering among patients with NCI and AD obtained using the N-AD1 (a), N-AD2 (b), and N-At (c) sets. S3 Fig. Validation of robustness of results of transcriptome data analysis. Dependencies of belonging to each subgroup of patients with AD based on the PDNA, PDAN, PtNA, and PtAN values (a). Dendrograms of the hierarchical clustering among patients with NCI and AD obtained using the N-A3 (b, upper panel) and N-A4 (b, lower panel) sets. S4 Fig. Results of epigenome data analysis. Jaccard coefficient of the genome-wide distributions of H3K27me3 (a), and CTCF (b) among individuals. Individuals were divided into two groups at the first divergence of the dendrogram. The epigenetic state exhibited by individuals belonging to the group containing the larger number of individuals was regarded as the “typical” epigenetic state, whereas that exhibited by individuals belonging to the group containing the fewer individuals was regarded as the “untypical” epigenetic state. Dashed lines indicate the boundaries between groups. The sum of squared errors (SSE) as a function of the number of clusters for the hierarchical clustering of individuals based on the Jaccard coefficient of the genome-wide distributions of H3K4me3 (see Fig. 4) (c). This plot shows the inferred number of clusters = 3. S5 Fig. Adjacency networks among substages. Adjacency network obtained using the M-NA and N-A1 sets (a) and that constructed using the M-NA and N-A2 sets (b). S6 Fig. Results of hierarchal clustering of patients with NCI and MCI and that of patients with MCI and AD. Examples of the hierarchal clustering of patients with NCI and MCI (a) and patients with MCI and AD (b). The sum of squared erro [file pone.0313733.s009.docx]

**SUPPORTING INFORMATION**

**S1 Table. Detailed information of analyzed data.** ID of individuals in this study, ID in the ENCODE database, diagnostic stage, inferred substage, inferred detailed substage, first, second, and third quantiles of MOR, availability of epigenome data, *APOE* genotype estimated using RNA-seq data (a). The results of the likelihood test by DEseq2, ANOVA, and the two-tailed *t*-test of expression levels of genes (b). Total number of reads from ChIP-seq data for H3K27ac, H3K27me3, H3K4me3, and CTCF mapped to the human genome (GRCh38) (c).

S1_Table.xlsx

**S2 Table. Detail features of M-NA set.** Gene list of M-NA set where the order of genes was the same as that in Fig 1b (a). Detailed results of the enrichment analysis were obtained using all genes in the M-NA set (b), those with higher expression levels in MCI_Major_ individuals (c), and AD risk genes with lower expression levels in MCI_Major_ individuals (d).

S2_Table.xlsx

**S3 Table. Detail features of N-AA and N-A2 set.** List of genes in the N-A_D1_ set (a), that in the N-A_D2_ set (b), that in the N-A_t_ set (c), that in the N-A1 set (d), and that in the N-A2 set (e). The order of genes in (d) and (e) are the same as that in Fig 3a and 3b, respectively. Genes in clusters CA1, CB1, CA2, and CB2 are also indicated. The P-values of ANOVA with the result of the Benjamini–Hochberg procedure (FDR < 0.1) of the expression level of each gene when the NCI stage is divided into three or four subgroups, and that of each gene among the three subgroups of AD, are also shown. Detailed results of the enrichment analysis were obtained using the genes in the N-A1 set (f), genes in the N-A1 set with higher expression levels in AD than in NCI (g), genes in the N-A1 set with lower expression levels in AD than in NCI (h), genes in the N-A2 set (i), genes in the N-A2 set with higher expression levels in AD than in NCI (j), and genes in the N-A2 set with lower expression levels in AD than in NCI (k). Detailed results of the enrichment analysis obtained using genes in clusters CA1 (l), CB1 (m), CA2 (n), and CB2 (o).

S3_Table.xlsx

**S4 Table. Detailed results of analysis of epigenome status and relationships between epigenome and transcriptome variations.** Values of Jaccard coefficients of genome wide maker peak regions among individuals (Upper) and density of marker for each chromosome of each individual (Lower) for H3K4me3 (a), H3K27ac (b), H3K27me3 (c), and CTCF (d). Correlation coefficient between expression levels of genes and H3K4me3 marker densities on promoters of these genes; correlation coefficient for common genes in CB1 and CB2 (Fig. 3), and that for common genes in CA1 and CA2 (Fig. 3) (e).

S4_Table.xlsx

**S5 Table. Matrix of confidence regarding the connections of adjacency networks among substages.** Matrix of confidence regarding the connections among patients with NCI, MCI_Major_, MCI_NCI_AD_, AD_HH_, AD_HL_, and AD_LL_ individuals estimated from the expression levels of the M-NA and N-A1 sets (a), and M-NA and N-A2 sets (b).

S5_Table.xlsx

**S6 Table. Matrix of confidence regarding the connections of adjacency networks among more detailed substages.** Matrix of confidence regarding the connections among the NCI_Typical_, NCI_MCI_, MCI_Typical,_ MCI_NCI_, MCI_AD_, MCI_NCI_AD_, AD_HH_MCI_, AD_HL_MCI_, AD_LL_MCI_, AD_HH_, AD_HL_, and AD_LL_ individuals estimated from the expression levels of the M-NA and N-A1 sets (a), and M-NA and N-A2 sets (b).

S6_Table.xlsx

**S7 Table. List of AD risk genes**. AD risk genes previously reported in the literature (Hu et al. 2017; Xiang et al. 2018; Rahman et al. 2019; Yang et al. 2022) where the order of genes was the same as that in S9 Fig. Many of these AD risk genes were obtained from studies using the hippocampus.

S7_Table.xlsx

**S8 Table. Significant DEGs among patients with NCI, MCI, and AD.**

S8_Table.xlsx

**S1 Fig.** **Dendrograms of the hierarchical clustering among the investigated individuals.** Dendrograms of the hierarchical clustering among individuals when P^D^_NMA_ = 0.01 (a), 0.02 (b), 0.03 (c), 0.04 (d), and 0.05 (e), and when P^A^_NMA_ = 0.01 (f), 0.02 (g), 0.03 (h), 0.04 (i), and 0.05 (j)

**S2 Fig. Dendrograms of hierarchical clustering among patients with NCI and AD.** Dendrograms of the hierarchical clustering among patients with NCI and AD obtained using the N-A_D1_ (a), N-A_D2_ (b), and N-A_t_ (c) sets.

**S3 Fig. Validation of robustness of results of transcriptome data analysis.** Dependencies of belonging to each subgroup of patients with AD based on the P^D^_NA_, P^D^_AN_, P^t^_NA,_ and P^t^_AN_ values (a). Dendrograms of the hierarchical clustering among patients with NCI and AD obtained using the N-A3 (b, upper panel) and N-A4 (b, lower panel) sets.

**S4 Fig. Results of epigenome data analysis.** Jaccard coefficient of the genome-wide distributions of H3K27me3 (a), and CTCF (b) among individuals. Individuals were divided into two groups at the first divergence of the dendrogram. The epigenetic state exhibited by individuals belonging to the group containing the larger number of individuals was regarded as the "typical" epigenetic state, whereas that exhibited by individuals belonging to the group containing the fewer individuals was regarded as the "untypical" epigenetic state. Dashed lines indicate the boundaries between groups. The sum of squared errors (SSE) as a function of the number of clusters for the hierarchical clustering of individuals based on the Jaccard coefficient of the genome-wide distributions of H3K4me3 (see Fig. 4) (c). This plot shows the inferred number of clusters = 3.

**S5 Fig. Adjacency networks among substages.** Adjacency network obtained using the M-NA and N-A1 sets (a) and that constructed using the M-NA and N-A2 sets (b).

**S6 Fig. Results of hierarchal clustering of patients with NCI and MCI and that of patients with MCI and AD.** Examples of the hierarchal clustering of patients with NCI and MCI (a) and patients with MCI and AD (b). The sum of squared errors (SSE) as a function of the number of clusters for the k-means clustering of MCI_Major_ individuals using the number of times each individual belonged to the NCI or AD clusters (c). This plot shows the inferred number of clusters = 3.

**S7 Fig. Results of elbow method to evaluate cluster number.** The sum of squared errors (SSE) as a function of the number of clusters for k-means clustering of patients with NCI using the number of times each individual belonged to the MCI cluster (a), and that of patients with AD using the number of times each individual belonged to the MCI cluster (b). The number of clusters = 2 was inferred in (a), while the number of clusters = 3 was inferred in (b).

**S8 Fig. Adjacency networks among more detailed substages.** Adjacency network obtained using the M-NA and N-A1 sets (a) and that constructed using the M-NA and N-A2 sets (b).

**S9 Fig. Results of the cluster analysis using AD risk genes reported by recent literatures.** Results of the cluster analysis of patients with NCI, MCI and AD (a) and that of patients with NCI and AD (b) using AD risk genes (S7 Table) previously reported in the literature (Hu et al. 2017; Xiang et al. 2018; Rahman et al. 2019; Yang et al. 2022). Many of these AD risk genes were obtained from studies using the hippocampus. The gene expression patterns of the hippocampus generally differs from that of the prefrontal cortex examined in this study. Thus, the results obtained in this study were quite different from those from previous studies.
